# Rupture of Heart*Read before the Buffalo Medical Association, February 5, 1884.

**Published:** 1884-02

**Authors:** C. C. Wyckoff


### Clinical Reports.

### Rupture of Heart.*

By Dr. C. C. Wyckoff.

I was called Nov. 30, 1883, to Mr. R., aged sixty-nine years. I found him pale, hands and feet cold, pulse 80, moderately full and regular. He had just come into the house after walking a short distance; complained of some dull pain in the centre of the chest, a little above the region of the nipples. I found that walking brought on a very sharp pain in this locality. At that time he had never had pain in either arm, nor more on one side of the chest than the other. He said that he had suffered from these attacks of pain for a year or more, and that they increased in frequency and severity of late, but with the exception of the paroxysms of pain he had felt very well for the last few months, yet acknowledged that he had lost flesh. Until quite recently he had supposed he was suffering from indigestion.

Dec. 1st. He went to his place of business and worked most of the day at book-keeping, but had some attacks of pain.

Dec. 2d, Sunday. Remained in the house; had several severe paroxysms of pain.

I saw him the second time, Dec. 3d. He was then having constant pain, with occasional severe paroxysms, as before, in the upper part of the centre of the chest, which were only controlled by inhalation of chloroform.

Dec. 4th. The pain extended from the centre of the chest, each way, and down the arms and hands to the ends of the fingers. After the paroxysms of severe pain subsided, he complained of pain upon the left side of chest, but of more severe pain under right shoulder blade.

Subcutaneous injections of morphia, ¼ grain, were given, which would make him comfortable for about six hours.

* Read before the Buffalo Medical Association, February 5, 1884.

Dec. 5th. Morphia still necessary to control the pain. Dr. Burwell in consultation at 3 P. M. As the bowels were constipated, enemas were resorted to without effect. Five grains of calomel were given, and followed in three hours by three grains more, to be followed in six hours from the first dose by Hunyadi water, which had to be repeated several times. However, the bowels were comfortably and satisfactorily moved in about twenty-four hours after the first dose of calomel; after which, Dec. 6th, morphia, subcutaneously, was repeated, which gave the patient a comfortable night.

Dec. 7th. No severe pain; morphia in the evening, after which there were no paroxysms of severe pain.

Dec. 8th and 9th. Patient, on the whole, very comfortable. He had slight pain over the region of the heart, accompanied with hyperæsthesia of the surface of chest in the same region.

I saw him last at about half-past five P. M., the 9th. At that time his pulse was 96, regular, and heart sounds normal; appeared comparatively comfortable, but complained of an uncomfortable feeling on the left side of the chest. He had eaten some solid food about one o'clock, for the first time during his confinement, and with a relish. In about five minutes after I left him, his wife, sitting at the foot of the bed, heard what she described as a "gurgling sound." She spoke to him, but received no answer; she noticed that his eyes were turned up, but there was no movement of any portion of the body. She called a lady from an adjoining room, after which he only gasped once.

*Autopsy:* Made by Dr. Frederick Peterson, eighteen hours after death. Body well nourished and well formed; rigor mortis slight; head not examined.

*Chest:* Lungs normal, with exception of slight emphysema and hypostatic congestion; pericardium contained about 250 cubic centimetres (or 8 oz.) of dark blood and coagula. There was a rupture of the heart from above downward, near the septum in the left ventricle, some six centimetres in length. The myocardium, to the naked eye, was extremely fatty, and under the microscope this fatty degeneration was corroborated, the fibres being almost destroyed. All about the place of rupture was deposited a thin layer of lymph upon the pericardium, showing that the rupture had been slowly going on for two or three days. There was no sign of endocarditis, nor of dissecting aneurism; no valvular lesions. The wall of the left ventricle seemed somewhat thinner at the point of rupture than elsewhere. Entangled in the edges of the ruptured spot, and protruding somewhat into the ventricular cavity, was a clot, dark and tough, of perhaps two or three days' existence. The coronary arteries were atheromatous to a great degree, and deforming endarteritis was also present in the aorta throughout its whole extent, and in the iliacs.

*Abdomen:* Spleen enlarged and hard, beginning cirrhosis of liver; right kidney weighed about 50 grammes; left was of normal size; both were hard, blue and granulous.

*Diagnosis:* Immediate cause of death, rupture of the heart, due to fatty degeneration and atrophy of part of the ventricular wall. Farther, chronic interstitial nephritis.

*Remarks:* Rupture of the heart most often occurs when there is, together with great general or partial fatty degeneration, a stenosis of the aortic valves. Such a case as this, where no aortic stenosis exists, is much more rare.

